# Essential Thrombocytosis in Patients <40 Years Old With Acute Coronary Syndromes: A Not So Uncommon Underlying Diagnosis Often Overlooked

**DOI:** 10.7759/cureus.32638

**Published:** 2022-12-17

**Authors:** Lisa Kok, Laura F Taverne, Eva C Verbeek, Machiel van de Wetering, Albertus J Voogel, Liane Oosterom, Jean-Paul R Herrman, Remko S Kuipers

**Affiliations:** 1 Cardiology, Onze Lieve Vrouwe Gasthuis, Amsterdam, NLD; 2 Cardiothoracic Surgery, Onze Lieve Vrouwe Gasthuis, Amsterdam, NLD; 3 Cardiology, BovenIJ Ziekenhuis, Amsterdam, NLD; 4 Cardiology, Spaarne Ziekenhuis, Haarlem, NLD; 5 Cardiology, Dijklander Ziekenhuis Purmerend, Purmerend, NLD

**Keywords:** traditional risk factors, myeloproliferative neoplasm, young adults, acute coronary syndrome, essential thrombocytosis

## Abstract

Background: In patients under <40 years, traditional cardiovascular (CV)-risk factors are a less likely cause of acute coronary syndromes (ACS) compared to older counterparts.

Aims: To estimate the prevalence of essential thrombocytosis (ET), a hematological disorder and less-prevalent risk factor, in young patients presenting with ACS.

Methods: We constructed a retrospective database of all patients <40 years (n=271) that had consecutively undergone coronary angiography (CAG) after their *first* ACS within our hospital within the last ten years (2010-2020) and had known thrombocyte counts (n=241). Patients with thrombocytes >450x10*9/L were screened for this hematological disorder.

Results: In our database, we identified 15 subjects with thrombocytosis. One was previously known as ET. Of the remaining 14 patients, five were considered reactive/secondary thrombocytosis, and four were lost to follow-up, four were eventually diagnosed with ET, one remains uncertain. The diagnosis was newly established before the initiation of this study in two patients (average delay: six years). Two patients were identified as a result of this study.

Conclusion: With a prevalence of at least 2.1%, ET appears not uncommon in patients <40 years with ACS. Moreover, screening patients with ACS *and* elevated thrombocytes yielded a novel diagnosis of ET in 27% of patients. The diagnosis was initially missed in all cases. Since the timing of revascularization should be adjusted to thrombocyte count/initiation of ET therapy to prevent thrombotic complications, cardiologists should know, recognize and screen for this pathology in ACS-patients, notably in those with absent traditional CV-risk factors: an ‘ACS-protocol’ aimed at less-prevalent risk factors could support this.

## Introduction

Traditional cardiovascular (CV)-risk factors explain most CV events [1]. Consequently, a reduced incidence of conventional risk factors will result in a relative increase in less-known risk factors [2-4]. Generally, the older the patient, the more likely an acute coronary syndrome (ACS) results from traditional risk factors [2,3]. Hence, the occurrence of ACS in patients <40 years and without conventional risk factors should alert the clinician to look for other causes of premature or accelerated atherosclerosis or increased thrombotic risk [1]. One such less-prevalent risk factor is a myeloproliferative neoplasm (MPN) [2,3].

As well known among hematologists, patients with one of the BCR/ABL-1-Philadelphia chromosome-negative MPNs, i.e., essential thrombocytosis (ET), polycythemia vera (PV) or primary myelofibrosis (PMF), have high incidences of CV-disease (CVD) [5]. The majority of the patients suffering from ET or PV die from CVD (26 and 25%, respectively) rather than from the disorder itself (13% each) [5]. Moreover, this increased relative risk of mortality from cardio- and cerebrovascular disease was especially noticed in younger patients. Most importantly, embolic complications often are the first manifestation of these hematological disorders [5].

As supported by pathological examinations of coronary arteries [6], cardiologists are faced with new challenges as the traditional ACS caused by a plaque rupture secondary to conventional risk factors is partly being replaced by other aetiologies caused by other, often less-prevalent, risk factors [4,6]. Our first research question was whether we could identify subjects with a less-prevalent risk factor, i.e., ET, by screening patients with thrombocytosis (i.e., a thrombocyte count >450x10^9^/L). Second, we wanted to compare the prevalence of ET in patients presenting with ACS with the prevalence reported 50 years ago (i.e., <1% [2]). Finally, we investigated whether ET was known, recognized, and screened for around ACS. We hypothesized that 1) we would be able to identify several cases of ET by screening notably young patients with thrombocytosis, 2) that the incidence of ET might be higher than previously reported, possibly due to a relative reduction in other risk factors, and that 3) less-prevalent risk factors for CV-disease, such as ET, are often overlooked. 

To test our hypotheses, we constructed a retrospective database of patients <40 years old who underwent coronary angiography (CAG) or percutaneous intervention (PCI) in the setting of an ACS. We chose a population <40 years since, in this group, the lowest exposure time to traditional and hence the highest prevalence of non-traditional risk factors was expected. The entire cohort was screened for elevated thrombocytes. In those patients with thrombocytosis, we differentiated between artifacts, reactive and essential thrombocytosis (according to WHO guidelines). Finally, we investigated how often an underlying ET was missed/noticed.

## Materials and methods

Study population

We constructed a database of all consecutive adults under the age of 40 years that underwent CAG/PCI in the setting of their first ACS in our referring hospital within the last 10 years (i.e., from 2010-2020). To collect these data, our local data manager created a query to isolate all patients that complied with the following criteria. 1. They should have undergone a CAG before 41 years, and 2. patients with a previous CAG were excluded. For all patients that fulfilled these criteria, laboratory results from the same admission (±one week) were screened for elevated (i.e.,>450x10^9^/L) thrombocytes. In all patients in whom elevated thrombocytes were found, additional laboratory results in the years following (ranging from 1-10 years, dependent on the moment of inclusion) were collected, if available. Additionally, peer communication was screened to determine whether elevated thrombocyte counts were noticed or paid attention to. The files of these patients were screened for a final diagnosis of MPN, ranging from PCV and PMF to ET. 

Additionally, the files and laboratory results of all patients in our database with platelet counts below 450x10^9^/L, i.e., those without elevated thrombocytes, during their initial visit were screened for elevated thrombocytes during subsequent visits, and terms like ‘thrombocytosis,’ ‘essential/primary thrombocytosis/thrombocythemia,’ ‘polycythemia vera’, ‘primary myelofibrosis’ to identify additional patients that had eventually developed an MPN, but had shown normal platelet counts around their CAG. 

In all cases, a diagnosis of either essential (primary) or reactive (secondary) thrombocytosis, or another MPN, had to be made/confirmed by a hematologist according to WHO guidelines. A patient had to fulfill all four primary criteria for a definite diagnosis of ET, i.e., 1. platelets >450x10^9^/L, 2. A characteristic bone marrow biopsy (with megakaryocyte proliferation and loose clusters), 3. Not meeting WHO criteria for other myeloid neoplasms, and 4. The presence of a JAK2/CALR/MPL mutation. Otherwise, the patient needed to fulfill the first three major and minor criterium, i.e., the presence of another clonal marker or no evidence of reactive thrombocytosis.

All patients considered suspects for an underlying MPN were screened for additional information. This information included basic anthropometrics ranging from age, sex, and concurrent cardiovascular risk factors to more specific facts such as the localization of the intervention, whether the MPN was previously known, the concurrent hemoglobin level and leukocyte counts, the drugs started after the coronary intervention, the type of cytoreduction started after the diagnosis of an MPN, the delay to the time of diagnosis of the MPN, the mutations found after genetic screening and the occurrence of complications during follow-up. The local Medical Ethical Committee approved the study under Ethics Approval number WO 21.045.

Study endpoints

The primary endpoint in our study was the number of patients (and the percentage of patients in our prespecified study population) that were found to have an underlying MPN that might have explained the ACS for which they underwent CAG.

Our secondary endpoints were 1. the identification of all patients with an elevated thrombocyte count, explained as artifacts, reactive or essential, 2. to find out whether elevated thrombocyte counts were noticed or paid attention to and 3. the time of the delay to diagnosis of an underlying MPN, in case it was initially missed.

## Results

**Figure 1:**
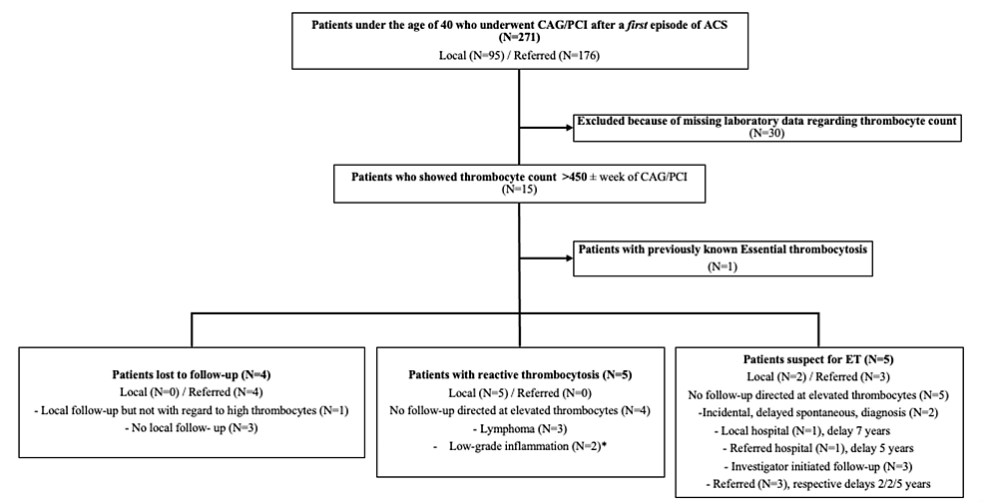
Flow chart of the study participants Flow chart showing in- and exclusions in the database, origin of the patients (local or referred from another hospital for intervention only), follow-up, and diagnosis delay. Abbreviations: CAG; coronary angiogram, PCI; percutaneous coronary intervention, ACS; acute coronary syndrome. * Low-grade inflammation due to recurrent smoking, obesity, or metabolic syndrome.

Between 2010 and 2020, 271 individuals (patients that underwent multiple angiographies were counted once) with ACS that underwent an invasive procedure were screened (Figure 1). Thirty patients were excluded due to absent thrombocyte counts. We identified 15 subjects with thrombocytosis (Table 1). One was previously known as ET. Five of the remaining 14 patients had reactive/secondary thrombocytosis, four were lost to follow-up, four were eventually diagnosed with ET, and one is pending since the patient refuses a bone marrow biopsy. Lost follow-up in patients with thrombocytosis was because they were immediately discharged to their referring hospital. Most were in transit in those referring hospitals and received no (local) follow-up (being either foreign or, e.g., homeless).

**Table 1:** Patient characteristics identified with elevated thrombocytes in our database of 241 patients that underwent CAG due to their first ACS. Abbreviations: CAG, coronary angiography, ACS, acute coronary syndrome; ET, essential thrombocytosis; MetS, metabolic syndrome; Invest Init, additional investigations into a possible diagnosis of ET were initiated because of this research project.

								Thrombo in letter	Thrombo follow-up
	Age	Sex	Other risk factors	Intervention	Hb	Leuko	Thrombocytes	Number/words	In lab
Previously known ET									
Case 1	30	M	Cannabis	PCI LAD	8.4	7.9	712	Yes/yes	Yes
Screened for ET after referral to a hematologist									
Case 2	39	M	Dyslipidemia	PCI-RCA	7.8	9.2	455	Yes/No	Yes, initially 365, later 550
Case 3	38	V	Diabetes type 1	Conservative	8.9	9.7	544	No/No	Yes, >450, not attributed to ET
Case 4	26	V	None	PCI LAD	9.4	15.7	592	Yes/No	No, Invest Init >450
Case 5	36	M	Smoking, obesity	PCI LAD	11.5	23.2	548	No/No	No, Invest Init >450
Case 6	39	M	Smoking	PCI LAD	8.8	10.3	519	No/No	No, incidental >450, Invest Init
Incomplete or lost to follow-up									
Case 7	40	V	None	PCI-RCX	6.3	10.6	502	Yes/No	Yes, 660
Case 8	30	M	Smoking	PCI LPL	10.6	17.5	519	Yes/No	No
Case 9	40	M	None	PCI RCA	9.5	31.6	510	Yes/No	No
Case 10	40	M	None	PCI-RCX	10.3	16.9	468	No/No	No
Considered reactive									
Case 11	40	V	Smoking, obesity, cannabis	PCI RCA	7	8.2	507	No/No	Yes, below 450
Case 12	37	V	Smoking, MetS	PCI RCA	9.1	10.7	467	No/No	Yes, below 400
Case 13	40	M	Malignancy	CABG-LIMA-LAD	7.2	7.6	569	No/No	Yes, below 400
Case 14	35	M	Malignancy	Conservative	6.4	6.5	539	Yes /No	Yes, below 400
Case 15	20	M	Malignancy	PCI LAD	8.8	8.3	805	Yes /No	Yes, below 400

Consequently, contact with these hospitals did not provide sufficient information to diagnose the presence/absence of primary/secondary ET. In the majority of our new cases of ET (i.e., 3), the elevated thrombocyte counts were copied into the discharge letter. However, in none of these discharge letters these divergent values were mentioned in the discussion/conclusion as being a possible/probable cause of the ACS.

The diagnosis (ET) was established through routine follow-up in two patients (average delay: six years). Two patients were newly identified as a result of this study. Details of the cases with thrombocytosis are depicted in (Table 2). Interestingly, one patient (Case 2) showed a normal platelet count (i.e., 365) on initial follow-up. Only seven years later, during routine follow-up, the thrombocytes amounted to >450 (i.e., 548), and the patient was diagnosed with a JAK2-positive ET. In another patient (Case 3), elevated thrombocytes were initially attributed to type 1 diabetes, and a JAK2-positive ET was delayed for 5 years. Two patients were diagnosed with ET as a result of this study. One diagnosis remains uncertain because of a lacking bone marrow biopsy.

**Table 2:** Patients suspect for ET, criteria for the diagnosis, and follow-up Abbreviations: DAPT: Double anti-platelet therapy; ASA, acetylsalicylic acid; +/- Charac BM, presence/absence of a characteristic bone marrow biopsy; +/- MPN, presence/absence of another myeloproliferative neoplasm; +/- mutation, presence/absence of a JAK2/CALR/MPL-mutation; +/- other clonal, presence/absence of another clonal marker; +/- reactive, presence or absence of (evidence of) reactive thrombocytosis.

			Drugs started after ACS	Follow-up	Cytoreduction	WHO-criteria for ET							
	Age	Sex	DAPT	Cytoreduction	Delay to ET	Since diagnosis	Charac BM	Other MPN	Mutation	Other clonal	Reactive	Mutation	Complications
							Major criteria	Minor criteria					
Previously known ET													
Case 1	30	M	ASA + ticagrelor	Hydrea	Known	Hydrea	+	-	+	-	-	JAK2	None
Newly diagnosed ET													
Case 2	39	M	ASA+ clopidogrel	None	7 years	Hydrea	+	-	+	-	-	JAK2	None
Case 3	38	V	ASA+ clopidogrel	None	5 years	Hydrea	+	-	+	-	-	JAK2	None
Case 4	26	V	ASA + ticagrelor	None	2 years	Hydrea	+	-	+	-	-	JAK2	None
Case 5	36	M	ASA + ticagrelor	None	2 years	Hydrea	+	-	-	-	-	Triple neg	None
Possible, but no definite ET													
Case 6	39	M	ASA + clopidogrel	None	5 years	None	Failed	-	-	-	-	Triple neg	None

After screening the files of all the remaining patients that had undergone CAG/PCI, we could not identify a single case with a late diagnosis of ET, PCV, or PMF. However, no specific laboratory or genetic screening was performed in this group which might result in an underestimation of the true prevalence of MPNs.

Long-term follow-up

During the follow-up period after stenting, none of the five patients eventually diagnosed with ET was readmitted for a recurrent ACS (Table 2), hence in our small subset, we do not have any indication for recurrent events, such as stent restenosis or stent thrombosis. However, only one (with previously known ET) out of our five patients with a (delayed) diagnosis of ET and ACS received hydroxyurea in the first year after the ACS.

## Discussion

In our final dataset of 241 consecutive patients, we identified 15 patients with thrombocytosis. Within this prespecified group, five patients proved to have either previously known or newly diagnosed ET. This amounts to 2.1% of patients with ACS and a prevalence of 33% of those patients with ACS and thrombocytosis. The prevalence might be higher since one patient with thrombocytosis (but absent mutations) refused a bone marrow biopsy, and four patients were lost to follow-up. No bone marrow biopsy or genetic testing was performed in patients with reactive thrombocytosis. In 27% of patients with ACS and thrombocytosis, ET could be newly diagnosed, which is in 1.7% of patients within our database.

Consequently, in these cases, the ACS was the first clear manifestation of their underlying MPN. Notably, the observation that the diagnosis (ET) was either delayed or missed in all cases might have had significant consequences. First, patients with ET who have suffered a CV event are classified as high-risk for recurrent events and hence require specific treatment with, e.g., hydroxyurea and (sometimes twice) daily aspirin to prevent future events [7]. Second, it has been argued that the timing of PCI/CABG in the setting of either stable angina or ACS as the first manifestation of ET should be adjusted to thrombocyte counts or the initiation of cytoreductive therapy to prevent peri-operative thrombotic complications [8-10]. Fortunately, our patients did not experience additional events during their follow-up periods (Table 2).

Although the incidence rate of ET is only about 1.5-2.5/100.000 per year [11,12] and ET is considered one of the rare disorders associated with ACS [2], the current data contest that ET is extremely rare when studying patients with ACS and a low exposure time to traditional CV-risk factors. In other words, ET is a rare disorder from a population perspective, but, as shown by our data, much higher rates (i.e., up to 2%) can be expected in specific clinical settings, such as in the practice of a neurologist, a vascular surgeon but also of a cardiologist, who all regularly see people with thrombi or thrombo-embolisms. In clinical practice, thrombotic/thrombo-embolic events are a frequent (up to 84%) first manifestation of ET [8,13-15], and cerebrovascular (55-56%) are more prevalent than either peripheral (13-22%) or coronary events (2-31%) in various ET-studies [15-18]. Interestingly, although thrombotic complications are a well-known complication of ET among hematologists, the current study suggests that ET is a much less-known underlying disorder for ACS among cardiologists. While the median age at diagnosis for ET is in the sixth decade of life [11,19], and less than 20% of patients are diagnosed below 40 years [20,21], the current study suggests that when appropriately screened, a subgroup of ET patients could be identified before the age of 40.

Importantly, ACS in ET patients has been observed in the presence and absence of underlying atherosclerosis since ET is known to induce both thrombosis and arterial narrowing due to chronic endothelial inflammation [22,23]. Consequently, as supported by this and other studies [22,24]: even very young patients without risk factors can experience acute, even life-threatening, thrombotic events if they have underlying ET [25]. Hence, we propose that, similar to an already existing ‘young stroke’ protocol for stroke patients <40 years and those of 40-50 years without traditional CV-risk factors, an ‘ACS protocol’ could be adopted for similar patients, i.e., those with low exposure to traditional CV-risk factors, presenting with ACS. Such patients should be actively screened for less-prevalent or known CV-risk factors [26]. Most, if not all, of these risk factors could be screened for with relatively little effort by a comprehensive anamnesis and physical examination, a prespecified laboratory analysis (including at least a hemoglobin, hematocrit, leukocyte, and platelet count, GFR, ASAT, ALAT, yGT, HDL, triglycerides, LDL, Lp(a), fasting/non-fasting glucose, HbA1c, CRP and TSH and possibly uric acid and homocysteine [27]), ambulatory rhythm monitoring and echocardiography (Table 3).

**Table 3:** ACS protocol for traditional and less-prevalent risk factors Abbreviations: LDL-c, low density lipoprotein cholesterol; HDL-c, high density lipoprotein cholesterol; TG, triglycerides; ESR, erythrocyte sedimentation rate; hsCRP, high sensitivity CRP; ASAT, aspartate aminotransferase; ALAT, alanine aminotransferase; yGT, gamma glutamyltransferase; ACS, acute coronary syndrome; CT, computed tomography;

Investigate traditional risk-factors	Possible testing
Smoking (including secondhand and, e.g., waterpipe, and e-cigarettes)	Anamnesis
Dyslipidemia	Laboratory testing (high LDL-c or high TG/low HDL-c)
Hypertension	Ambulatory monitoring
Diabetes mellitus	Laboratory testing (screen glucose or HbA1c)
Family history (first-degree male < 55, female < 65 years)	Anamnesis
Screen for causes of premature/accelerated atherosclerosis	
Drugs (e.g., cocaine, nitrous oxide, marihuana)	Urine/blood testing
Inflammatory/immune diseases	Inflammatory markers (e.g., ESR, hsCRP, Leukocytes/thrombocytes)
Malignancies (e.g., hematological malignancies such as ET)	Laboratory testing (e.g., total blood count, ESR)
Metabolic syndrome	Laboratory testing (e.g., ASAT, ALAT, yGT, TG, HDL-C)
Consider other underlying etiologies for ACS	
Atrial fibrillation	Ambulatory rhythm monitoring
Aortic thrombi	Echocardiography
Cardiomyopathy (e.g., Tako-Tsubo)	Echocardiography
Coronary artery dissection	Coronary angiography
Coronary abnormalities	CT-angiography
Deep vein thrombosis (paradoxical embolism)	Echography
Drugs (e.g., cocaine, nitrous oxide)	Urine/blood testing
Endocarditis	Echocardiography
Inflammatory/immune diseases	Inflammatory markers (e.g., ESR, hsCRP, Leukocytes/thrombocytes)
Intracardiac tumors	Echocardiography
Malignancies (e.g., hematological malignancies such as ET)	Laboratory testing (e.g., total blood count, ESR)
Coronary vasospasm (e.g., menses, partus of menopause-related)	Coronary angiography

Interestingly, about such laboratory parameters, the applicable Guidelines from the European Society of Cardiology (either STEMI (2018), Chronic Coronary Syndromes (CCS, 2019), NSTEMI (2020) or CVD prevention (2021)) do contain some advice about prognostic testing, i.e., a full blood count (CCS, 2019; CVD, 2021), renal function, liver function (in hypertensives only, CVD 2021), lipid profile, fasting plasma glucose and HbA1c (as part of diabetes screening) and thyroid function (CCS, 2019), but do not refer to such tests as being part of ‘screening for underlying disorders in ACS’. Moreover, although a total blood count should include a leukocyte and platelet count, neither leukocytes nor platelets are mentioned as CV-risk factors in these guidelines, despite several extensive studies showing the association between hematological malignancies -with either elevated thrombocytes or leukocytes [7,11,28], and ACS/CCS.

Next, the cut-off value of >450x10^9^/L for thrombocytosis screening could be a matter of debate since, in one of our ET patients, a subsequent value was as low as 365x10^9^/L (without treatment). A study that screened for JAK2-V617F on a population base [29] showed a low (i.e., 23%) sensitivity for this mutation to predict a future MPN. However, this sensitivity increased to >90% when hemoglobin (>10 mmol/L), leukocyte (>7x10^9^/L), and platelet counts (>350x10^9^/L) were taken into a screening algorithm [30]. Consequently, in patients with ACS, notably those with negligible traditional CV-risk factors, subsequent screening for ET, i.e., at least repetitive thrombocyte counts, could be considered from a platelet count > 350x10^9^/L.

Finally, the current study clearly showed that despite the presence of both elevated thrombocytes and a thrombotic event, the diagnosis of ET was either delayed or missed. Taken together, from the facts that the elevated thrombocytes were often visible within the discharge letters but rarely described as being connected to the ACS, one can conclude that ET might be insufficiently known or recognized. Hence, a standardized protocol for screening for risk factors (see Table 3) should be used in ACS patients. Furthermore, every hospital discharge letter should include all investigations undertaken, notably those that were abnormal. These letters should consist of a discussion or conclusion regarding (the differential diagnosis of the) underlying pathology for the ACS and advice for further screening.

Limitations

Limitations of our study are the facts that it was conducted retrospectively, that we did not have thrombocyte counts of all our patients, that about 25% of our patients with thrombocytosis were lost to follow-up and that we did not perform genetic testing in our whole database, so we do not have information on the presence of, e.g., a JAK2-mutation in ACS-patients without thrombocytosis.

## Conclusions

When screening only those patients with an elevated platelet count (i.e.,>450x10*9/L), ET could be established in at least 33% of patients <40 years undergoing CAG/PCI in the setting of ACS. Hence, ET is a not-so-uncommon (around 2%) underlying disorder in these patients. Moreover, in most cases, the ACS was their first manifestation of underlying ET and, thus, the first opportunity to start adequate preventive treatment.
